# Pathological neural networks and artificial neural networks in ALS: diagnostic classification based on pathognomonic neuroimaging features

**DOI:** 10.1007/s00415-021-10801-5

**Published:** 2021-09-28

**Authors:** Peter Bede, Aizuri Murad, Orla Hardiman

**Affiliations:** 1 Computational Neuroimaging Group, Trinity Biomedical Sciences Institute, Trinity College Dublin, Room 5.43, Pearse Street, Dublin 2, Ireland; 2 Pitié-Salpêtrière University Hospital, Sorbonne University, Paris, France

**Keywords:** Neuroradiology, Machine learning, Amyotrophic lateral sclerosis, Neuroimaging, Artificial neural networks

## Abstract

The description of group-level, genotype- and phenotype-associated imaging traits is academically important, but the practical demands of clinical neurology centre on the accurate classification of individual patients into clinically relevant diagnostic, prognostic and phenotypic categories. Similarly, pharmaceutical trials require the precision stratification of participants based on quantitative measures. A single-centre study was conducted with a uniform imaging protocol to test the accuracy of an artificial neural network classification scheme on a cohort of 378 participants composed of patients with ALS, healthy subjects and disease controls. A comprehensive panel of cerebral volumetric measures, cortical indices and white matter integrity values were systematically retrieved from each participant and fed into a multilayer perceptron model. Data were partitioned into training and testing and receiver-operating characteristic curves were generated for the three study-groups. Area under the curve values were 0.930 for patients with ALS, 0.958 for disease controls, and 0.931 for healthy controls relying on all input imaging variables. The ranking of variables by classification importance revealed that white matter metrics were far more relevant than grey matter indices to classify single subjects. The model was further tested in a subset of patients scanned within 6 weeks of their diagnosis and an AUC of 0.915 was achieved. Our study indicates that individual subjects may be accurately categorised into diagnostic groups in an observer-independent classification framework based on multiparametric, spatially registered radiology data. The development and validation of viable computational models to interpret single imaging datasets are urgently required for a variety of clinical and clinical trial applications.

## Introduction

Diagnostic delay in neurodegenerative conditions has a considerable literature. In ALS, the average interval from symptom onset to definite diagnosis is around 12 months [1, 2]. Patients often describe insidious symptom onset many months before medical advice is sought. The key milestones of the diagnostic journey in ALS include symptom manifestation, visit to a general practitioner, review in a general neurology clinic, diagnostic investigations, and assessment in a tertiary referral centre to confirm a suspected diagnosis [3–6]. The constellation of initial symptoms may be confounded by comorbid conditions, and misdiagnoses in the initial phase of ALS are not uncommon. The implications of diagnostic delay are considerable as it may delay recruitment into clinical trials, may have ramifications for genetic counselling, may increase the risk of misdiagnoses or potentially lead to unnecessary medical or surgical interventions such as spinal laminectomies, carpal tunnel surgery, and intravenous immunoglobulin (IVIg) treatment [1]. Recent imaging studies have revealed that by the time the diagnosis is confirmed, significant neurodegenerative changes have already occurred [7], limiting the potential of putative neuroprotective medications. Recent evidence also suggests that considerable presymptomatic disease burden can be readily detected long before symptom manifestation [8–11]. These observations would suggest that, the optimal window for clinical trials is not well into the ‘post-diagnostic’ phase of the disease, when widespread cerebral and spinal cord degeneration can already be detected, but as early as the diagnostic likelihood or mutation status permit. The role of neuroimaging in ALS has been extensively discussed [12], but the literature is dominated by papers describing group-level, phenotype- or genotype-associated imaging traits [13]. Various research consortia have invested considerable effort to increase cohort numbers, pool data from multiple centres to perform well-powered analyses and report radiological patterns representative of a particular phenotype [14]. The characterisation of stereotyped ‘signatures’ is academically interesting [12, 15], but the practical demands of clinical practice are markedly different [16]. As opposed to the scholarly pursuit of group-level descriptions, the priority of clinical neurology is the precision classification of a specific, single patient into diagnostic, phenotypic and prognostic categories through the quantitative interpretation of their biomarker profile. Relatively few studies have focussed on the classification of individual patient imaging data in ALS [17, 18]. A variety of innovative approaches have been explored [19] spanning from z-score based approaches, through support vector machine frameworks, discriminant function analyses, to regression models, with varying degree of classification accuracy [16, 20–24]. Several studies have reported excellent ‘area under the curve’ (AUC) values with reference to the discriminatory potential of a specific measure between patients and healthy controls, but binary classification into ‘ALS’ versus ‘healthy’ does not mirror real-life diagnostic dilemmas. In the clinical setting, the distinction between ‘ALS’ and ‘healthy’ is seldom challenging; instead, the dilemma is typically whether subtle clinical changes represent incipient ALS or rather, the harbinger of an alternative neurodegenerative condition. Another common shortcoming of classification studies is the a priori selection of anatomical regions, often referred to as ‘regions of interest’ (ROIs) which are known to be affected in ALS, rather than performing formal feature selection analyses or ranking variables based on their discriminatory potential. Finally, few studies have narrowed their analyses to a cohort of patients in their peri-diagnostic phase, which seems indispensable to scrutinise and validate proposed frameworks. The classification of cases with marked disability and long disease duration reveals relatively little about the efficacy of a specific model architecture. Accordingly, the objective of this study is the development of an observer-independent, multiclass (three-way) classification protocol to categorise multiparametric imaging data of a large cohort of subjects consisting of patients with amyotrophic lateral sclerosis (ALS), healthy controls (HC) and disease controls (DC). An additional objective of the study is to evaluate and rank the importance of imaging measures and anatomical foci for further model optimisation, and to test a proposed classification framework on subjects in their peri-diagnostic phase.

## Methods

### Participants

A total of 378 participants, 214 patients with ALS (‘ALS’), 37 disease controls (‘DC’) with a non-ALS neurodegenerative diagnosis and 127 healthy controls (‘HC’) were included in a prospective, single-centre imaging study. All participants gave informed consent in accordance with the Ethics Approval of this research project (Beaumont Hospital, Dublin, Ireland). Exclusion criteria included prior cerebrovascular events, known traumatic brain injury, comorbid neoplastic, paraneoplastic or neuroinflammatory diagnoses. Participating ALS patients were diagnosed according to the revised El Escorial criteria. Disease controls consisted of patients with FTD and were diagnosed based on the Rascovsky criteria. Participating patients had a uniform neurological assessment and key variables, such as disability scores, interval from diagnosis to scan, and handedness were recorded.

### Magnetic resonance imaging

A standardised imaging protocol was implemented on a 3 Tesla Philips Achieva Magnetic resonance (MR) platform. A 3D Inversion Recovery prepared Spoiled Gradient Recalled echo (IR-SPGR) sequence was utilised to acquire T1-weighted (T1w) images with a field-of-view (FOV) of 256 × 256 × 160 mm, flip angle = 8°, spatial resolution of 1 mm^3^, SENSE factor = 1.5, TR/TE = 8.5/3.9 ms, TI = 1060 ms. A spin-echo echo planar imaging (SE-EPI) pulse sequence was used to acquire diffusion tensor images (DTI) using a 32-direction Stejskal-Tanner diffusion encoding scheme; FOV = 245 × 245 × 150 mm, 60 slices with no interslice gap, spatial resolution = 2.5 mm^3^, TR/TE = 7639/59 ms, SENSE factor = 2.5, *b* values = 0, 1100 s/mm^2^, dynamic stabilisation and spectral presaturation with inversion recovery (SPIR) fat suppression. To assess for comorbid inflammatory or vascular pathologies, fluid-attenuated inversion recovery (FLAIR) images were also acquired from each subject. An Inversion Recovery Turbo Spin Echo (IR-TSE) sequence was used for FLAIR imaging. Data were acquired in axial orientation: FOV = 230 × 183 × 150 mm, spatial resolution = 0.65 × 0.87 × 4 mm, 30 slices with 1 mm gap, TR/TE = 11,000/125 ms, TI = 2800 ms, 120° refocusing pulse, with flow compensation and motion smoothing and a saturation slab covering the neck region.

### Imaging framework

Initial quality control steps included radiological review for incidental pathological findings, assessment for movement artefacts, and evaluation of white matter abnormalities on FLAIR. Following standardised pre-processing steps (described below), 28 volumetric metrics, 68 cortical thickness values and 120 white matter indices were uniformly retrieved from each subject’s imaging data; a total of 216 imaging measures were then appraised in each participant. These data were systematically analysed in post-hoc statistics.

### Volume metrics

The standard anatomical reconstruction pipeline of the FreeSurfer image analysis suite [25], ‘recon-all’ was implemented, including non-parametric non-uniform intensity normalisation, affine registration to the MNI305 atlas, intensity normalisation, skull striping, automatic subcortical segmentation, linear volumetric registration, neck removal, tessellation of the grey matter-white matter boundary, surface smoothing, inflation to minimise metric distortion, and automated topology correction [26]. To segment the brainstem into the medulla oblongata, pons and midbrain, a Bayesian segmentation algorithm was utilised, which relies on a probabilistic atlas of the brainstem and its neighbouring anatomical structures generated based on 49 scans [27]. The following 28 cerebral volume values were uniformly retrieved from each pre-processed T1-weighted dataset: (1) left cerebellar white matter volume, (2) left cerebellar cortex volume, (3) left thalamus volume, (4) left caudate volume, (5) left putamen volume, (6) left pallidum volume, (7) left hippocampus volume, (8) left amygdala volume, (9) left accumbens volume, (10) right cerebellar white matter volume, (11) right cerebellar cortex volume, (12) right thalamus volume, (13) right caudate volume, (14) right putamen volume, (15) right pallidum volume, (16) right hippocampus volume, (17) right amygdala volume, (18) right accumbens volume, (19) posterior corpus callosum volume, (20) middle corpus callosum volume, (21) central corpus callosum volume, (22) mid-anterior corpus callosum volume, (23) anterior corpus callosum volume, (24) medulla volume, (25) pons volume, (26) superior cerebellar peduncle volume, (27) midbrain volume, and (28) total intracranial volume. Volumetric values of individual subjects were converted as percentage of the subject’s total intracranial volume (TIV) to account for individual TIV variations.

### Cortical thickness values

Following pre-processing with ‘recon-all’, the labels of the Desikan–Killiany atlas were utilised to retrieve average cortical thickness values [20] from 34 cortical regions in the left and right cerebral hemispheres; (1) banks superior temporal sulcus, (2) caudal anterior-cingulate cortex, (3) caudal middle frontal gyrus, (4) cuneus cortex, (5) entorhinal cortex, (6) frontal pole, (7) fusiform gyrus, (8) inferior parietal cortex, (9) inferior temporal gyrus, (10) insula, (11) isthmus–cingulate cortex, (12) lateral occipital cortex, (13) lateral orbitofrontal cortex, (14) lingual gyrus, (15) medial orbital frontal cortex, (16) middle temporal gyrus, (17) parahippocampal gyrus, (18) paracentral lobule, (19) pars opercularis, (20) pars orbitalis, (21) pars triangularis, (22) pericalcarine cortex, (23) postcentral gyrus (24) posterior-cingulate cortex, (25) precentral gyrus, (26) precuneus cortex, (27) rostral anterior-cingulate cortex, (28) rostral middle frontal gyrus, (29) superior frontal gyrus, (30) superior parietal cortex, (31) superior temporal gyrus, (32) supramarginal gyrus, (33) temporal pole, and (34) transverse temporal cortex.

### White matter indices

Pre-processing of diffusion tensor data were implemented using in FMRIB’s software library. Raw DTI data first underwent eddy current corrections and skull removal; a tensor model was then fitted to generate maps of axial diffusivity (AD), fractional anisotropy (FA), mean diffusivity (MD), and radial diffusivity (RD). FMRIB’s software library’s tract-based statistics (TBSS) module was utilised for non-linear registration and skeletonisation of individual DTI images. A mean FA mask was created and each subject’s individual AD, FA, MD and RD images were merged into 4-dimensional (4D) AD, FA, MD and RD image files. The study-specific white matter skeleton was masked by atlas-defined labels for the following 30 white matter regions of interests in MNI space: (1) left anterior thalamic radiation, (2) right anterior thalamic radiation, (3) left cerebellar white matter skeleton averaged, (4) right cerebellar white matter skeleton averaged, (5) left cingulum, (6) right cingulum, (7) left corticospinal tract, (8) right corticospinal tract, (9) left external capsule, (10) right external capsule, (11) forceps major, (12) forceps minor, (13) fornix, (14) left inferior cerebellar peduncle, (15) right inferior cerebellar peduncle, (16) left inferior fronto-occipital fasciculus, (17) right inferior fronto-occipital fasciculus, (18) left inferior longitudinal fasciculus, (19) right inferior longitudinal fasciculus, (20) left medial lemniscus, (21) right medial lemniscus, (22) middle cerebellar peduncle, (23) left posterior thalamic radiation, (24) right posterior thalamic radiation, (25) left superior cerebellar peduncle, (26) right superior cerebellar peduncle, (27) left superior longitudinal fasciculus, (28) right superior longitudinal fasciculus, (29) left uncinate fasciculus, and (30) right uncinate fasciculus. The labels of the standard-space ICBM-DTI-81 white matter atlas [28, 29] were utilised to create masks for the cerebellar peduncles, medial lemniscus, external capsule and posterior thalamic radiation. Labels of the JHU white matter tractography atlas [30, 31] were used to generate masks for the forceps major and minor, anterior thalamic radiation, uncinate, superior and inferior longitudinal fasciculi, cingulum, corticospinal tracts, inferior fronto-occipital fasciculi. The cerebellar label (label 2) of the MNI probabilistic atlas [32, 33] was used to generate a mask for averaged cerebellar diffusivity estimation. The FMRIB fornix template [34] was used to mask the study-specific white matter skeleton in MNI space. Four diffusivity metrics (AD, FA, MD, RD) were retrieved from 30 white matter regions in each subject, resulting in a white matter panel of 120 values.

### Statistical analyses

An artificial neural network framework, a multilayer perceptron model was implemented with hyperbolic tangent as the hidden layer activation function. The diagnosis (ALS, HC, DC) was set as dependent variable, and the retrieved imaging measures as covariates. Imaging metrics were rescaled by standardisation; (*x* − mean)/s. The model architecture included one hidden layer with 6 units. Data were partitioned into a training sample (68%) and testing sample (32%). A batch-type training approach was utilised with a gradient descent optimisation algorithm; initial learning rate: 0.4, momentum: 0.9, interval centre: 0, interval offset: ± 0.5. Using the above model architecture, the following outputs were generated; synaptic weights, classification results, ROC curves, and AUC values. An independent variable importance analysis was also performed to rank the relevance of imaging metrics in determining group membership. To visually represent the accuracy of diagnostic classification, the predicted pseudo-probability of each diagnostic group was plotted in a bar chart. Based on the feature importance hierarchy, a streamlined classification model was tested using only the 20 most important imaging variables. Finally, to further scrutinise the classification framework, the model was tested on a subset of patients in their peri-diagnosis phase, who were scanned within 6 weeks of their formal diagnosis.

## Results

**Fig. 1:**
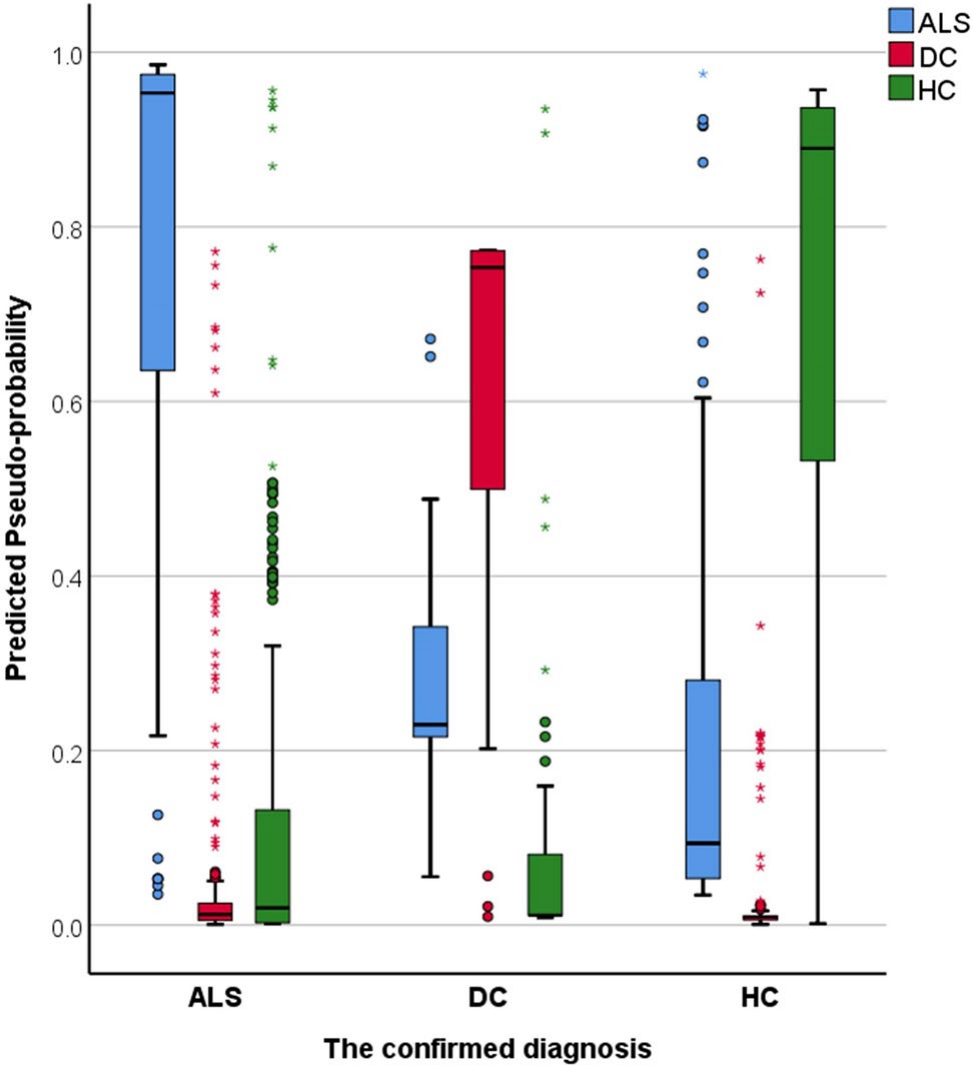
The predicted pseudo-probability profiles of subjects with an established diagnosis

**Fig. 2:**
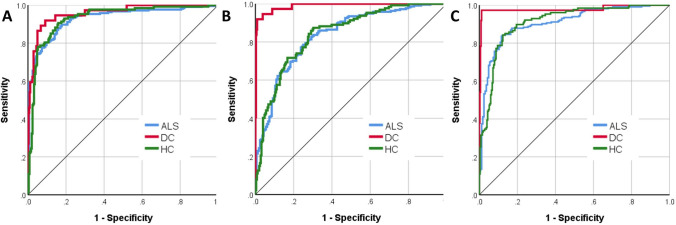
Receiver-operating characteristic curve of patients with ALS, disease controls (DC) and healthy controls (HC) using all imaging features (**A**—left), the 20 most important variables only (**B**—middle) and white matter diffusivity metrics alone (**C**—right)

**Fig. 3:**
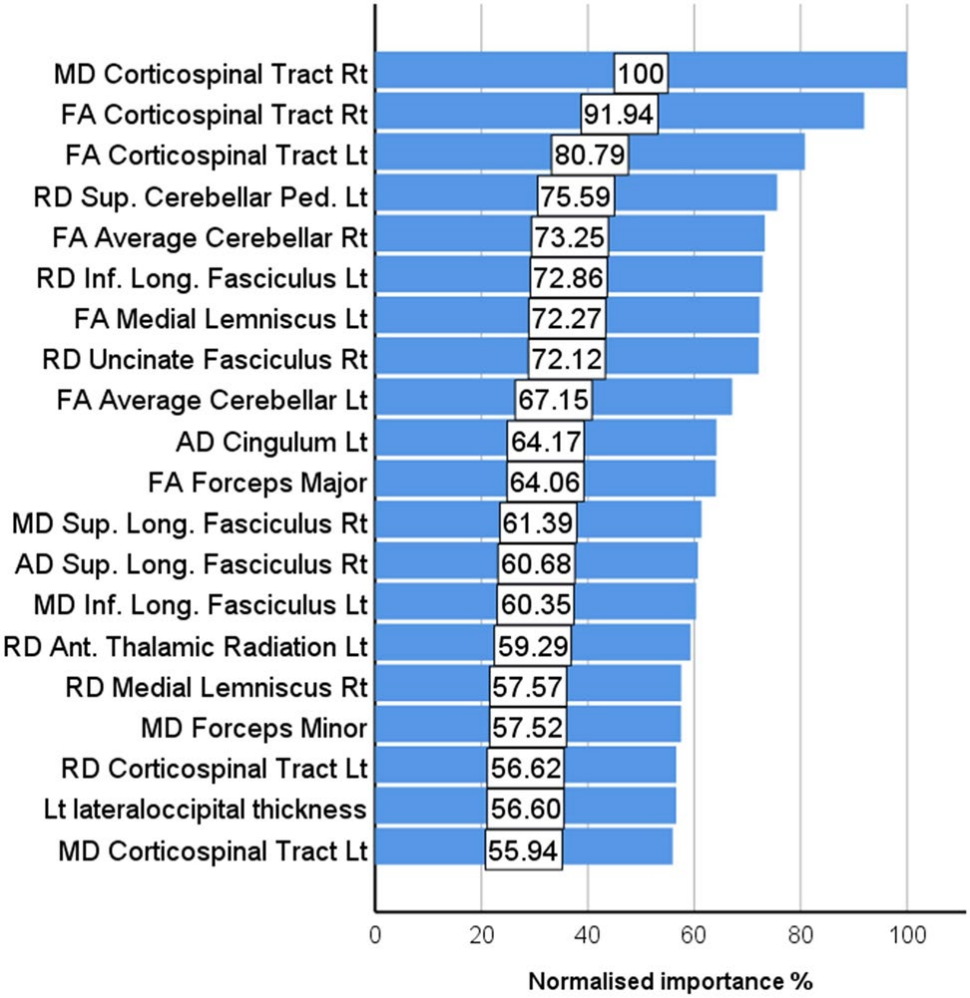
The hierarchy of normalised variable importance

The three groups, ALS (*n* = 214, age: 60.97 ± 11.92, 140 male, 202 right handed), healthy controls (*n* = 127, age: 59.29 ± 10.95, 59 male, 112 right handed) and disease controls (*n* = 37, age: 62.4 ± 7.9, 20 male, 34 right handed) were matched for age (*p* = 0.23) and handedness (*p* = 0.12), but not for sex (*p* = 0.002). Of the 378 datasets, 256 (67.7%) was included in the training sample and 122 (32.3%) in the testing sample. Cross-entropy error was 73.49 in the training sample and 85.03 in the testing sample; incorrect predications were 10.9% in the training sample and 24.6% in the testing sample. Classification summary is presented in Table 1A. The predicted pseudo-probability of diagnosis in each cohort (confirmed diagnosis) is presented in Fig. 1. Receiver-operating characteristic (ROC) curves are presented in Fig. 2A. Area under the curve values were 0.930 for ALS, 0.958 for disease controls, and 0.931 for healthy controls relying on all input imaging variables. The normalised importance of the 20 most relevant imaging variables in predicting group membership is shown in Fig. 3 with their corresponding importance value. The ranked normalised importance of the 50 most relevant imaging metrics is presented in Table 2. The classification analyses were re-run with the 20 most important imaging metrics identified by the explorative analyses. Relying on only 20 imaging features, the classification accuracy was evaluated again (Table 1B). Area under the curve values based on only 20 core imaging features (Fig. 2B) were 0.835 for ALS, 0.990 for DC, and 0.842 for healthy controls. As 19 white matter metrics were ranked among the 20 most important imaging features (Fig. 3) and the vast majority (92%) of imaging metrics among the 50 diagnostically relevant variables (Table 2) were diffusivity metrics, a final post hoc analysis was conducted where only white matter diffusivity indices were included as covariates in the perceptron model; all 30 tracts and all four diffusivity metrics (120 variables in total). Area under the receiver-operating characteristic curves generated based on white matter features alone (Fig. 2C) were 0.907 for ALS, 0.979 for DC, and 0.911 for healthy controls. Classification outcomes using white measures alone are presented in Table 1C.

**Table 1:** Classification outcomes in the training and testing samples using A, all imaging features B, the 20 most important variables only and C, white matter diffusivity metrics alone

Sample	Observed	Predicted			
		ALS	DC	HC	% Correct
(A) All features included					
Training	ALS	140	2	9	92.7
	DC	3	19	2	79.2
	HC	11	1	69	85.2
	Overall percent	60.2%	8.6%	31.3%	89.1
Testing	ALS	52	6	5	82.5
	DC	2	9	2	69.2
	HC	14	1	31	67.4
	Overall percent	55.7%	13.1%	31.1%	75.4
(B) 20 core features included					
Training	ALS	126	1	32	79.2
	DC	3	23	1	85.2
	HC	24	0	63	72.4
	Overall percent	56.0%	8.8%	35.2%	77.7
Testing	ALS	46	0	9	83.6
	DC	2	7	1	70.0
	HC	15	0	25	62.5
	Overall percent	60.0%	6.7%	33.3%	74.3
(C) Only white matter metrics included					
Training	ALS	128	1	18	87.1
	DC	0	24	0	100.0
	HC	16	0	70	81.4
	Overall percent	56.0%	9.7%	34.2%	86.4
Testing	ALS	53	3	11	79.1
	DC	0	12	1	92.3
	HC	11	0	30	73.2
	Overall percent	52.9%	12.4%	34.7%	78.5

*ALS* amyotrophic lateral sclerosis, *DC* disease control, *HC* healthy control

**Table 2:** The importance and normalised importance of the 50 most relevant imaging variables in predicting group membership

Rank	Imaging metric	Importance	Normalised importance (%)
1.	MD Corticospinal Tract Rt	0.014	100.0
2.	FA Corticospinal Tract Rt	0.013	91.9
3.	FA Corticospinal Tract Lt	0.011	80.8
4.	RD Sup Cerebellar Ped Lt	0.010	75.6
5.	FA Average Cerebellar Rt	0.010	73.3
6.	RD Inf. Longitudinal_Fasciculus Lt	0.010	72.9
7.	FA Medial Lemniscus Lt	0.010	72.3
8.	RD Uncinate Fasciculus Rt	0.010	72.1
9.	FA Average Cerebellar Lt	0.009	67.1
10.	AD Cingulum Lt	0.009	64.2
11.	FA Forceps Major	0.009	64.1
12.	MD Sup. Longitudinal Fasciculus Rt	0.008	61.4
13.	AD Sup. Longitudinal Fasciculus Rt	0.008	60.7
14.	MD Inf. Longitudinal Fasciculus Lt	0.008	60.4
15	RD Anterior Thalamic Radiation Lt	0.008	59.3
16.	RD Medial Lemniscus Rt	0.008	57.6
17.	MD Forceps Minor	0.008	57.5
18.	RD Corticospinal Tract Lt	0.008	56.6
19.	Lt lateraloccipital thickness	0.008	56.6
20.	MD Corticospinal Tract Lt	0.008	55.9
21.	RD Medial Lemniscus Lt	0.008	55.6
22.	AD External Capsule Rt	0.008	54.6
23.	FA Uncinate Fasciculus Rt	0.007	54.2
24.	RD Sup. Cerebellar Ped Rt	0.007	54.0
25.	Lt posteriorcingulate thickness	0.007	53.7
26.	AD Forceps Minor	0.007	53.6
27.	RD Anterior Thalamic Radiation Rt	0.007	53.5
28.	FA Inf. Cerebellar Peduncle Rt	0.007	53.1
29.	FA Inf. Longitudinal Fasciculus Rt	0.007	52.4
30.	Lt precentral thickness	0.007	51.1
31.	RD Average Cerebellar Lt	0.007	51.0
32.	RD Inf. Cerebellar Peduncle Lt	0.007	51.0
33.	MD Middle Cerebellar Peduncle	0.007	50.7
34.	RD Inf. Cerebellar Peduncle Rt	0.007	50.1
35.	FA Inf. Cerebellar Peduncle Lt	0.007	49.9
36.	MD Post. Thalamic Radiation Rt	0.007	49.8
37.	MD Inf. Fronto-Occipital Fasciculus Rt	0.007	49.7
38.	FA Inf. Longitudinal Fasciculus Lt	0.007	49.3
39.	FA Sup. Cerebellar Peduncle Lt	0.007	48.8
40.	MD Medial Lemniscus Lt	0.007	48.3
41.	Lt Caudal middle frontal thickness	0.007	48.2
42.	AD Medial Lemniscus Rt	0.007	47.4
43.	Left-Amygdala Volume %	0.007	47.1
44.	RD Middle Cerebellar Peduncle	0.007	47.0
45.	MD Anterior Thalamic Radiation Rt	0.006	46.8
46.	FA Uncinate Fasciculus Lt	0.006	45.7
47.	FA Medial Lemniscus Rt	0.006	45.4
48.	RD Forceps Major	0.006	45.3
49.	RD Forceps Minor	0.006	45.0
50.	MD External Capsule Rt	0.006	45.0

*AD* axial diffusivity, *ALS* amyotrophic lateral sclerosis, *DC* disease control, *FA* fractional anisotropy, *HC* healthy control, *Lt* left, *MD* mean diffusivity, *RD* radial diffusivity, *Rt* right

**Fig. 4:**
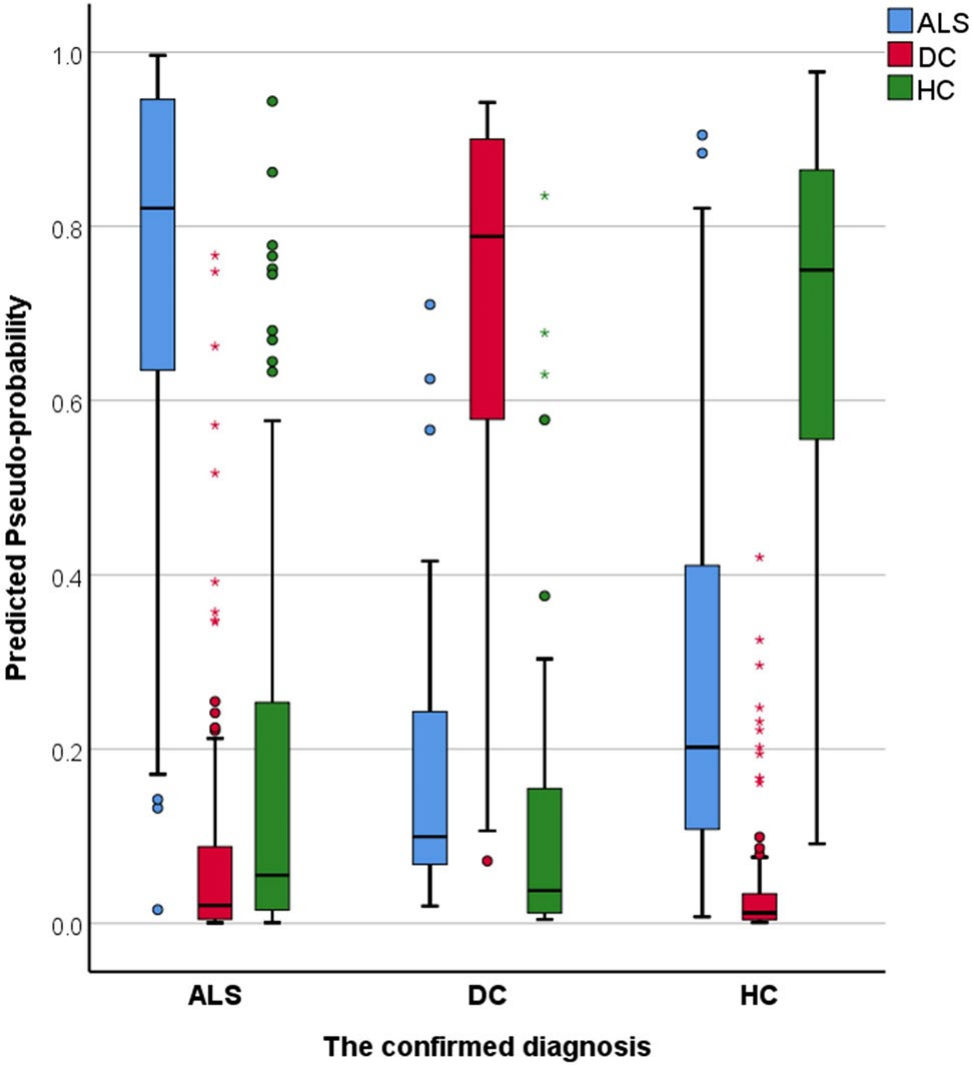
The predicted pseudo-probability profiles of ALS patients around the time of their diagnosis (< 6 weeks), disease controls (DC) and healthy controls (HC)

**Fig. 5:**
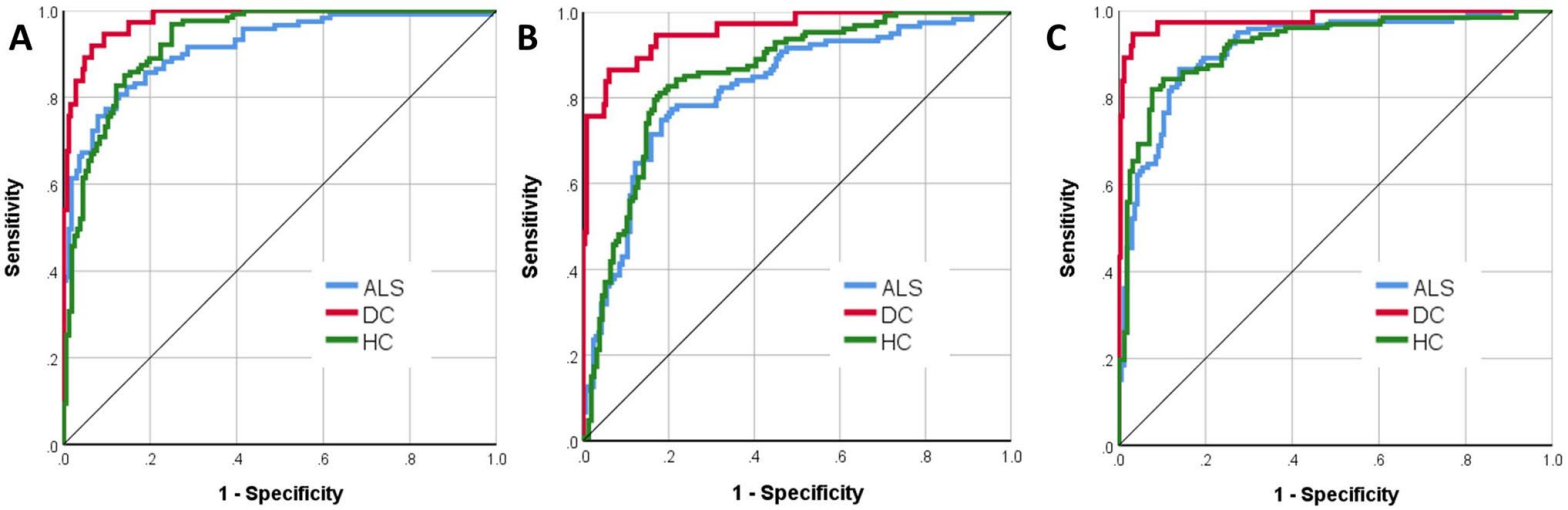
Receiver-operating characteristic curve of patients with ALS around the time of their diagnosis (< 6 weeks), disease controls (DC) and healthy controls (HC) using all imaging features (**A**—left), the 20 most important variables only (**B**—middle) and white matter diffusivity metrics alone (**C**—right)

**Fig. 6:**
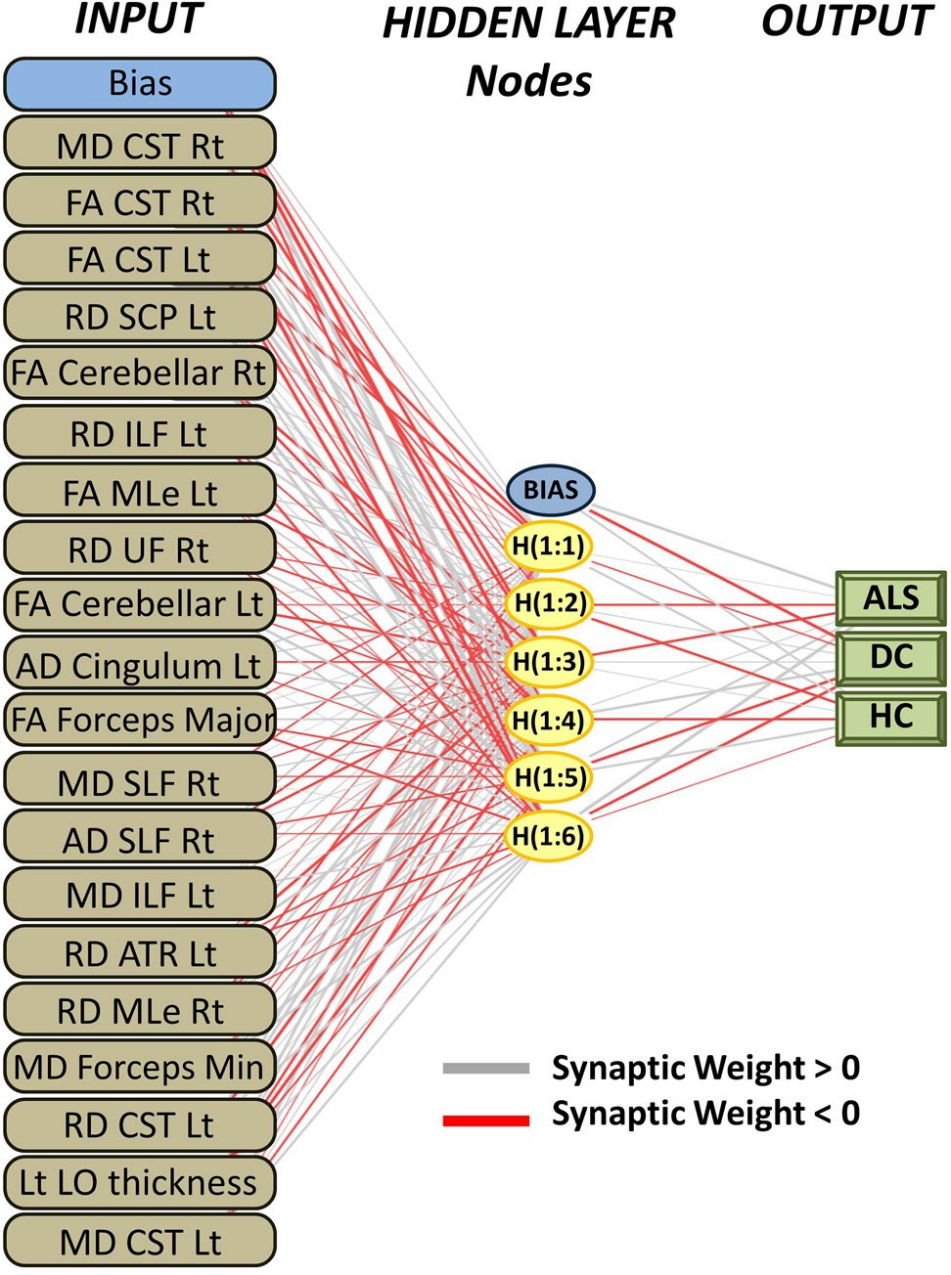
The multilayer perceptron model architecture. Input layer: 20 imaging metrics. Hidden layer: 6 nodes (units). Hidden layer activation function: hyperbolic tangent. Output layer: diagnostic label. *CST* corticospinal tract, *SCP* sup cerebellar ped, *ILF* inferior longitudinal fasciculus, *MLe* medial lemniscus, *UF* uncinate fasciculus, *SLF* superior longitudinal fasciculus, *ATR* anterior thalamic radiation, *LO* lateral occipital, *Rt* right, *Lt* left

To scrutinise the validity of this classification strategy, the model was tested on a subset of patients (*n* = 119) who were scanned within 6 weeks of their diagnoses (‘peri-diagnosis cohort’). Using all imaging features AUC was 0.915 for ALS, 0.979 for DC and 0.929 for HC. Using the 20 most important imaging features alone, AUC was 0.822 for ALS, 0.958 for DC and 0.853 for HC. Using all WM metrics but no GM measures, AUC was 0.914 for ALS, 0.981 for DC and 0.92 for HC. Classification outcomes in the ‘peri-diagnosis’ cohort are presented in Table 3. Pseudo-probability profiles in the peri-diagnostic phase using all imaging features are presented in Fig. 4 and the three ROC curves are shown in Fig. 5. Model architecture is presented in Fig. 6 with 20 input variables.

**Table 3:** Classification outcomes in the peri-diagnostic phase in the training and testing samples using A, all imaging features B, the 20 most important variables only and C, white matter diffusivity metrics alone

Sample	Observed	Predicted			
		ALS	DC	HC	% Correct
(A) All features included					
Training	ALS	76	5	9	84.4
	DC	1	19	3	82.6
	HC	15	1	65	80.2
	Overall percent	47.4%	12.9%	39.7%	82.5
Testing	ALS	23	0	6	79.3
	DC	2	11	1	78.6
	HC	11	0	35	76.1
	Overall percent	40.4%	12.4%	47.2%	77.5
(B) 20 features included					
Training	ALS	58	3	24	68.2
	DC	3	22	1	84.6
	HC	15	1	68	81.0
	Overall percent	39.0%	13.3%	47.7%	75.9
Testing	ALS	26	4	4	76.5
	DC	1	6	4	54.5
	HC	7	0	36	83.7
	Overall percent	38.6%	11.4%	50.0%	77.3
(C) Only white matter metrics included					
Training	ALS	62	3	13	79.5
	DC	1	24	0	96.0
	HC	12	1	75	85.2
	Overall percent	39.3%	14.7%	46.1%	84.3
Testing	ALS	30	2	9	73.2
	DC	2	9	1	75.0
	HC	5	1	33	84.6
	Overall percent	40.2%	13.0%	46.7%	78.3

*ALS* amyotrophic lateral sclerosis, *DC* disease control, *HC* healthy control

## Discussion

Our data indicate that quantitative imaging aids diagnostic classification and the systematic assessment of key anatomical regions may not only help to distinguish ALS from healthy controls, but also discriminates it from other neurodegenerative conditions. The presented framework operates in an observer-independent fashion and receiver-operating characteristic curves indicate excellent sensitivity/specificity profiles. In addition to the classification accuracy of the multilayer perceptron model utilised, the ranking of imaging features with respect to categorisation relevance offers valuable insights for the streamlining and optimisation of future models.

The utility of a variety of supervised and unsupervised machine-learning approaches have been explored in ALS, including support vector machines, regression-based approaches, random forests, discriminant function analyses, dimension reduction frameworks, but these are seldom applied to imaging data [17, 35, 36] due to challenges associated with MRI scanning, quality control, pre-processing, data acquisition costs and data harmonisation. Advanced neural network architectures have been successfully trialled in other conditions, including multilayer ‘deep-learning’ learning models and generative adversarial networks (GAN) [37–40]. The development of automated diagnostic frameworks based on radiology data in ALS are hampered by the scarcity of large, uniformly acquired training data sets. While the acquisition and recording of epidemiology data and clinical measures can be relatively easily harmonised, imaging data harmonisation requires considerable investment.

Our study consisted of an exploratory arm, where imaging metrics from the entire cerebrum were incorporated, without the a priori selection of anatomic regions considered relevant based on published imaging or post mortem evidence. While the predilection of disease burden to the corticospinal tracts, precentral gyrus and brainstem is well established, our strategy centred on the indiscriminate interrogation of imaging variables from across the entire cerebrum. To develop a truly observer-independent pipeline, individual imaging data were spatially co-registered to standard space, and only validated atlases were utilised to retrieve integrity variables from a range of anatomical regions. Cortical and subcortical, grey matter and white matter, supratentorial and infratentorial, left and right hemisphere structures were uniformly evaluated without prioritising potentially discriminatory anatomical regions a priori.

The ranking of variable importance revealed interesting trends. By large, integrity metrics of white matter regions discriminated the three groups better than grey matter measures. This is in line with previous observations that white matter degeneration is a relatively early feature of ALS [7, 41], while GM changes are less consistent, and may only become readily detectable in the later stages of the disease. A practical ramification of the recognition of the superior discriminatory power of white matter measures is that diffusion tensor protocols should be routinely incorporated into clinical and pharmacological trials protocols as opposed to only relying on T1-weighted, FLAIR and T2-weighted data sets which are classically used for clinical evaluation to rule out mimic conditions. Interestingly, there was only one grey matter variable among the first 20 diagnostically relevant imaging features and only 4 grey matter variables were ranked in the first 50 features. Our post hoc analyses also confirmed that excellent subject classification can be achieved relying on white matter measures alone (Figs. 2 and 5, Tables 1 and 3). These models provided accurate diagnostic classification without evaluating grey matter measures or volumes at all, and were solely based on measures derived from DTI. Furthermore, our results confirm the imperative of evaluating non-FA diffusivity measures. While FA is the most commonly evaluated white matter metric in descriptive analyses, RD, MD, and AD proved to be equally important discriminatory variables in our models. The review of ranked discriminating variables (Table 2.) is not only interesting from the perspective of biophysical measures, but also from an anatomical standpoint. The relative importance of key ALS-associated brain regions such as corticospinal tracts and precentral gyrus is not surprising given the ample evidence of the pathognomonic involvement of these structures in ALS. Conversely, the indices of some brain regions, such as the brainstem, ranked relatively low in the hierarchy of feature importance despite their archetypal involvement in ALS [42]. The discriminatory relevance of external capsule integrity is also of interest as ALS studies overwhelmingly emphasise internal capsule alterations [43]. It is also noteworthy that multiple cerebellar measures are among the most important discriminatory features, including intra-cerebellar white matter diffusivity metrics, volumetric values as well as cerebellar peduncle integrity measures. The recognition that cerebellar degeneration is an important facet of ALS biology is not new, but regional cerebellar disease burden has only been recently characterised in detail [44–50]. Our findings highlight the practical importance of systematically evaluating infratentorial indices in ALS and not only focussing on supratentorial variables. Several long association tracts (ILF, FOF) were also listed among the first 50 discriminatory regions, which are likely to aid discrimination from the disease-control group [51]. Frontotemporal dementia is a genetically, molecularly and clinically heterogeneous group of conditions, and specific subtypes are associated with specific imaging signatures [52, 53]. Our study illustrates the relevance of assessing brain regions which are not classically affected in ALS [54]. These regions may be preferentially affected in other conditions therefore the interrogation of imaging metrics from these anatomical foci is invaluable in discriminating ALS from alternative diagnoses. More broadly, our results support the importance of exploring imaging data without a priori anatomical assumptions.

Our approach illustrates that a multitude of metrics may be readily incorporated into complex classification models across a variety of anatomical regions. These models may be potentially further expanded to include additional measures such as wet biomarkers, additional imaging metrics or clinical measures [55–64]. In this application only cerebral measures were evaluated, despite the potential of spinal metrics [65–68]. Similar frameworks could potentially be utilised for the discrimination of other MND phenotypes such as PLS, PMA or flail-arm syndrome [69–71].

This study is not without limitations. A three-way classification scheme was implemented with a single disease-control group. The inclusion of a ‘mimic’ disease-control group would have been helpful to test the model further, but the definition of a true ALS mimic condition is contentious. Only total volumes of subcortical structures were explored as input variables in this study, even though the assessment of specific amygdalar nuclei, thalamic nuclei or hippocampal subfields may enhance the discrimination of ALS form other neurodegenerative conditions [72–75]. Moreover, our model only evaluated cerebral metrics, therefore LMN pathology is not accounted for and discrimination from LMN-predominant MNDs cannot be reliably assessed [76–82]. Additional validation of the model with presymptomatic mutation carriers would have tested the classification accuracy of the model further by evaluating subjects with limited disease burden [10, 83]. Model overfitting to a particular training cohort is invariably a significant risk and this study is no exception. Notwithstanding these limitations, our results indicate that subjects may be accurately classified into a diagnostic cohort, healthy control, or diseases control categories based on imaging data alone.

## Conclusions

The meaningful interpretation of singe-subject imaging data is an urgent priority of clinical neuroradiology. Group-level descriptive analyses offer valuable academic insights, but the practical demands of clinical neuroradiology and clinical trial applications require accurate single-subject classification based on a core set of quantitative markers.

## Data Availability

Personal medical data are not publically available due to institutional data regulations.

## Abbreviations

AD: Axial diffusivity
ALS: Amyotrophic lateral sclerosis
ALSFRS-r: Revised amyotrophic lateral sclerosis functional rating scale
ANN: Artificial neural network
ATR: Anterior thalamic radiation
AUC: Area under the curve
*C9orf72*: Chromosome 9 open reading frame 72
CST: Corticospinal tract
CT: Cortical thickness
DC: Disease control
DTI: Diffusion tensor imaging
EMM: Estimated marginal mean
EPI: Echo-planar imaging
FA: Fractional anisotropy
FLAIR: Fluid-attenuated inversion recovery
FOF: Fronto-occipital fasciculus
FOV: Field of view
FTD: Frontotemporal dementia
FWE: Family-wise error
GAN: Generative adversarial networks
GM: Grey matter
HC: Healthy control
ICP: Inferior cerebellar peduncle
ILF: Inferior longitudinal fasciculus
IR-SPGR: Inversion recovery prepared spoiled gradient recalled echo
IR-TSE: Inversion recovery turbo spin-echo sequence
IVIG: Intravenous immunoglobulin
LMN: Lower motor neuron
LO: Lateral occipital
Lt: Left
MCP: Middle cerebellar peduncle
MD: Mean diffusivity
ML: Machine learning
MLe: Medial lemniscus
MND: Motor neuron disease
MNI152: Montreal Neurological Institute 152 standard space
MPM: Multilayer perceptron model
NISALS: Neuroimaging Society in ALS
PBA: Pseudobulbar affect
PCC: Pathological crying and laughing
PMC: Primary motor cortex
QC: Quality control
RD: Radial diffusivity
ROC: Receiver-operating characteristic curve
ROI: Region of interest
Rt: Right
SCP: Superior cerebellar peduncle
SD: Standard deviation
SE-EPI: Spin-echo echo planar imaging
SENSE: Sensitivity encoding
SLF: Superior longitudinal fasciculus
SPIR: Spectral presaturation with inversion recovery
T1w: T1-weighted imaging
TBSS: Tract-based spatial statistics
TE: Echo time
TFCE: Threshold-free cluster enhancement
TI: Inversion time
TIV: Total intracranial volume
TR: Repetition time
UF: Uncinate fasciculus
UMN: Upper motor neuron
WM: White matter

## Code availability

Statistical procedures are described in the methods section of the manuscript.
